# Identification of Five N6-Methylandenosine-Related ncRNA Signatures to Predict the Overall Survival of Patients with Gastric Cancer

**DOI:** 10.1155/2022/7765900

**Published:** 2022-04-08

**Authors:** Qingfang Yue, Yuan Zhang, Jun Bai, Xianglong Duan, Haipeng Wang

**Affiliations:** ^1^Department of Medical Oncology, Shaanxi Provincial People's Hospital, Xi'an, 710068 Shaanxi, China; ^2^Institute of Medical Research, Northwestern Polytechnic University, Xi'an, 710072 Shaanxi, China; ^3^Second Department of General Surgery, Shaanxi Provincial People's Hospital, Xi'an, 710068 Shaanxi, China; ^4^Medical College, Xizang Minzu University, Xianyang, 712082 Shaanxi, China

## Abstract

Noncoding ribonucleic acids (ncRNAs) are involved in various functions in the formation and progression of different tumors. However, the association between N6-methyladenosine-related ncRNAs (m6A-related ncRNAs) and gastric cancer (GC) prognosis remains elusive. As such, this research was aimed at identifying m6A-related ncRNAs (lncRNAs and miRNAs) in GC and developing prognostic models of relevant m6A-related ncRNAs and identifying potential biomarkers regulated by m6A. In this study, the m6A2Target database, Starbase database, and The Cancer Genome Atlas (TCGA) were used to screen m6A-related ncRNAs. And then, we performed integrated bioinformatics analyses to determine prognosis-associated ncRNAs and to develop the m6A-related ncRNA prognostic signature (m6A-NPS) for GC patients. Finally, five m6A-related ncRNAs (including lnc-ARHGAP12, lnc-HYPM-1, lnc-WDR7-11, LINC02266, and lnc-PRIM2-7) were identified to establish m6A-NPS. The predictive power of m6A-NPS was better in the receiver operating characteristic (ROC) curve analysis of the training set (area under the curve (AUC), >0.6). The m6A-NPS could be utilized to classify patients into high- and low-risk cohorts, and the Kaplan-Meier analysis indicated that participants in the high-risk cohort had a poorer prognosis. The entire TCGA dataset substantiated the predictive value of m6A-NPS. Significant differences in TCGA molecular GC subtypes were observed between high- and low-risk cohorts. The ROC curve analysis indicated that m6A-NPS had better predictive power than other clinical characteristics of GC prognosis. Uni- and multivariate regression analyses indicated m6A-NPS as an independent prognostic factor. Furthermore, the m6A status between the low-risk cohort and high-risk cohort was significantly different. Differential genes between them were enriched in multiple tumor-associated signaling pathways. In summary, five m6A-related ncRNA signatures that could forecast the overall survival of patients with GC were identified.

## 1. Introduction

The current global statistic reveals that gastric cancer (GC) is the third leading contributor of cancer-related mortality, with an incidence that widely varied across regions, i.e., >70% in developing countries, mainly in East Asia [1]. In China, GC is identified as the second-highest risk factor of cancer-related mortality, ranking second among common malignancies in men and third in women [2]. GC is influenced by various major risk factors, such as poor dietary habits, active smoking, and *Helicobacter pylori* infection. Due to genetic heterogeneity and early screening difficulties [3, 4], the prognosis of patients with GC remains unsatisfactory, especially in China [5]. Therefore, effective biomarkers should be identified to better assess tumor progression, predict the overall survival (OS), and improve treatment outcomes.

As one of the most common chemical modifications of eukaryotic messenger ribonucleic (mRNA), N6-methyladenosine (m6A) can affect various essential biological processes by regulating the expression of target genes [6, 7]. m6A-regulated proteins consist of “writers” (WTAP, METTL3, and METTL14), “erasers” (ALKBH5 and FTO), and “readers” (IGF2BPs and YTHDFs) [8–10]. Existing evidence has demonstrated that m6A modifications contribute to a vital function in regulating the maturation, translation, and degradation of precursor mRNAs. Several studies have shown that m6A regulator dysregulation is associated with apoptosis, proliferation, self-renewal, developmental defects, and malignant tumor progression [11–15].

Noncoding RNAs (ncRNAs) are transcripts with no potential for protein coding and include small ncRNAs (sncRNAs, 18–200 nt) as well as long ncRNAs (lncRNAs, >200 nt). Various ncRNAs types include microRNAs (miRNAs), ribosomal RNAs (rRNAs), small nucleolar RNAs (snoRNAs), transfer RNAs (tRNAs), and long noncoding RNAs (lncRNAs). The majority of ncRNAs participate in different cellular processes including apoptosis, proliferation, cell cycle, epithelial-mesenchymal transition, and autophagy [16]. Among these ncRNAs, miRNAs and lncRNAs are known to regulate gene expression by modifying the underlying transcriptional mechanism or through their fine regulation at different levels, such as transcription, translation, and protein function. In GC, aberrant expression of ncRNAs is strongly linked to tumor progression, radioresistance, chemoresistance, and sensitivity to target therapy or immunotherapy [17–21]. Among them, the aberrant expression of miRNAs has been well investigated in gastric cancer. For example, Deng et al. found that high expression of oncogenic miR-215 in GC tissues might be a promising biomarker for GC diagnosis [22]. Zheng et al. reported that miR-148a is considered to be one of the important tumor suppressors in GC and linked to lymph node metastasis and TNM staging [23]. A plethora of evidence also suggested that aberrant expression of lncRNAs serve as tumor suppressors or carcinogens in the development of GC. Recently, several lncRNAs are aberrantly expressed in GC and tightly linked to prognosis; for example, HOTAIR was reported as an oncogenic lncRNA that can promote proliferation and invasion by multiple mechanisms and its high expression significantly linked to poor prognosis of GC patients [19, 24, 25]. lncRNA-PVT1 was reported that its overexpression in GC tissues can promote cell proliferation by regulating the expression of FOXM1, p15, and p16 and significantly associated with poor overall survival [26].

Recently, accumulating evidence suggests that m6A modification plays a key role for regulating a range of bioprocesses, including ncRNA processing and their biological function in tumorigenesis [27, 28]. Some ncRNAs involved in different types of cancer were simultaneously shown to acquire dynamic m6A modifications in their structures, such as XIST, MALAT1, and HOTAIR [29]. Recently, Zhang et al. reported that ALKBH5 (an eraser enzyme) can promote GC invasion and metastasis by demethylating the lncRNA NEAT1 [30]. Another study has indicated that the m6A demethylase ALKBH5 could inhibit the lncRNA PVT1 degradation, and its overexpression facilitated the proliferation of osteosarcoma cells *in vitro* and *in vivo* [31]. Chen et al. revealed that METTL14 suppressed colorectal carcinogenesis via regulating m6A-dependent primary miR-375 processing [32]. A similar phenomenon showed that METTL14 could inhibit colorectal cancer progression by downregulating the oncogenic lncRNA XIST [33]. The lncRNA GAS5 has been reported to inhibit colorectal cancer progression by regulating the Yes-associated protein phosphorylation and degradation and to be negatively modulated by the m6A reader YTHDF3 [34]. However, the potential prognostic value of m6A-associated ncRNAs in GC and the function of ncRNAs associated with m6A regulators in GC remains unknown. Therefore, it is of utmost importance to investigate biomarkers that can be used as potential therapeutic targets from the perspective of the mechanisms of m6A modifications.

At present, methods for repurposing microarray data for ncRNA expression analysis have been well-established [35, 36]. Hence, this research was aimed at identifying m6A-related ncRNAs (lncRNAs and miRNAs) in GC and developing prognostic models of relevant m6A-related ncRNAs and identifying potential biomarkers regulated by m6A.

## 2. Materials and Methods

### 2.1. Data Collection

Transcriptome data of GC patient samples were obtained from The Cancer Genome Atlas (TCGA). Stomach cancer dataset and the matching clinical survival information were downloaded. Based on the clinical data, transcriptome samples with complete survival information were obtained. Finally, an aggregate of 347 samples was screened for prognostic gene expression profiles and prognostic model development. Furthermore, 21 m6A-related genes include “writers” (ZC3H13, VIRMA (KIAA1429), WTAP, RBM15, RBM15B, METTL3, METTL14, and METTL16), “erasers” (ALKBH5 and FTO), and “readers” (IGF2BP1, IGF2BP2, IGF2BP3, RBMX, HNRNPC, HNRNPA2B1, YTHDC1, YTHDC2, YTHDF1, YTHDF2, and YTHDF3) [37].

### 2.2. Acquisition of m6A-Related ncRNAs

m6A-related ncRNAs were identified utilizing the Pearson correlation analysis (with the ∣Pearson *R* | >0.3 and *p* < 0.01) in the TCGA dataset. m6A2Target database (http://m6a2target.canceromics.org/) was employed to screen m6A-related ncRNAs and mRNAs, sequentially. Then, the Starbase database (http://starbase.sysu.edu.cn/index.php) was utilized to screen m6A-related mRNA-interacting ncRNAs. Finally, these datasets were merged to obtain candidate m6A-related ncRNAs, which were analyzed in the subsequent analysis.

### 2.3. Development of Weighted Gene Coexpression Networks

The expression matrices of 347 samples were categorized randomly into the training and testing sets (including training set (*n* = 243) and testing set (*n* = 104)). A coexpression network was created for the ncRNA expression matrix using the R package “WGCNA.” A total of six relevant modules were obtained, brown and blue were selected as key modules according to the patients' survival status, and a total of 910 ncRNAs were obtained as candidate genes.

### 2.4. Establishing a Prognostic Model Based on m6A-Related ncRNAs

In the TCGA-GC training set, the univariate Cox regression analysis was executed on candidate genes to detect prognosis-related genes, with *p* < 0.001 as the screening threshold. Then, the least absolute shrinkage and selection operator (LASSO) Cox regression analysis was utilized to further assess prognostic genes. Therefore, prognostic gene signatures were developed according to the LASSO Cox regression model coefficients (*β*-values) multiplied by ncRNA expression levels. The signature risk score of each sample was computed as follows: Risk score = *β*ncRNA_1_∗ncRNA_1_ + *β*ncRNA_2_∗ncRNA_2_ + ⋯+*β*ncRNA_*n*_∗ncRNA_*n*_ expressions. Then, the risk score was computed for each participant in the training set, and the samples were categorized as high- or low-risk cohorts. Time-dependent receiver operating characteristic (ROC) curves were utilized to determine the model's predictive power for the 1-, 3-, and 5-year survivals. The model's prognostic ability was evaluated utilizing the Kaplan-Meier log-rank tests. The efficacy of the model is corroborated utilizing the testing set and the entire TCGA dataset to analyze its survival and ROC curves.

### 2.5. Performance Assessment of m6A-NPS

*T*-test analysis was performed for the comparison of risk score differences according to clinical characteristics. ROC curves were employed to compare the predictive ability of the risk score model with other clinical features to determine the GC prognosis. The uni- and multivariate Cox regression analyses were carried out to validate the model independence.

### 2.6. Functional Enrichment Analyses

The expression profile of 21 m6A-related genes in low- and high-risk cohorts was analyzed. Moreover, differentially expressed genes (DEGs) were detected between low- and high-risk cohorts utilizing the R package “Deseq2” with the criterion of log2 (fold change)  | >1.5 and *p* < 0.05), and then, DEGs were entered into the “Metascape” website (https://metascape.org/gp/index.html) for functional enrichment analysis [38].

## 3. Results

### 3.1. Identification of m6A-Related ncRNAs in GC

**Figure 1:**
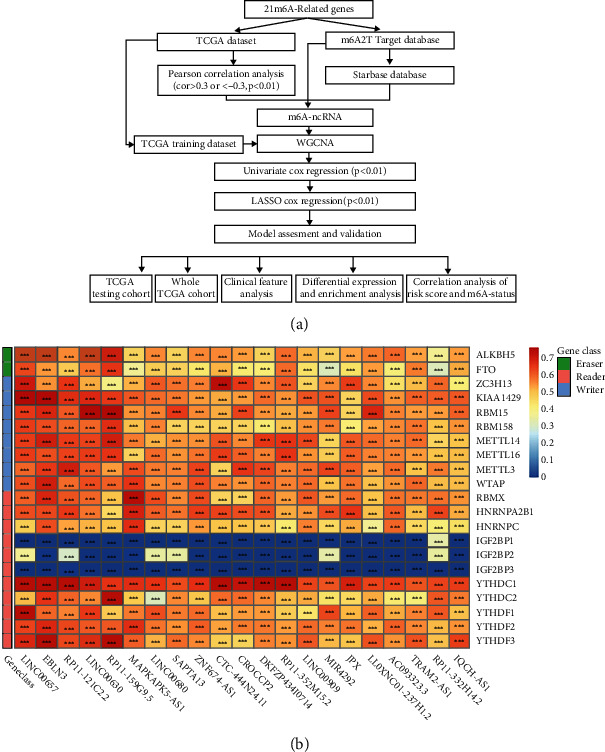
(a) The workflow analysis of this study. (b) Heatmap of the correlations between m6A-related genes and 21 prognostic m6A-related ncRNAs. ^∗^*p* < 0.05, ^∗∗^*p* < 0.01, and ^∗∗∗^*p* < 0.001.

**Figure 2:**
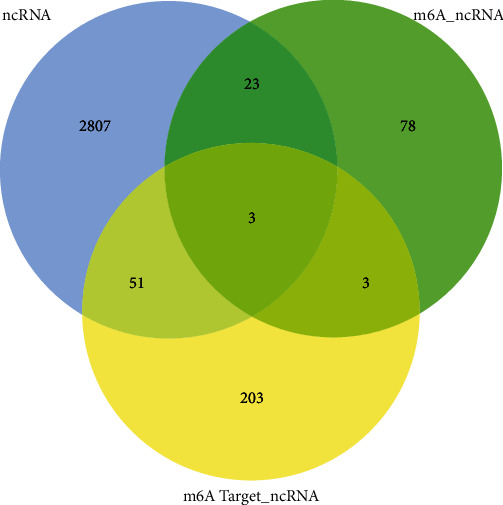
Venn diagram of m6A-related ncRNAs.

**Figure 3:**
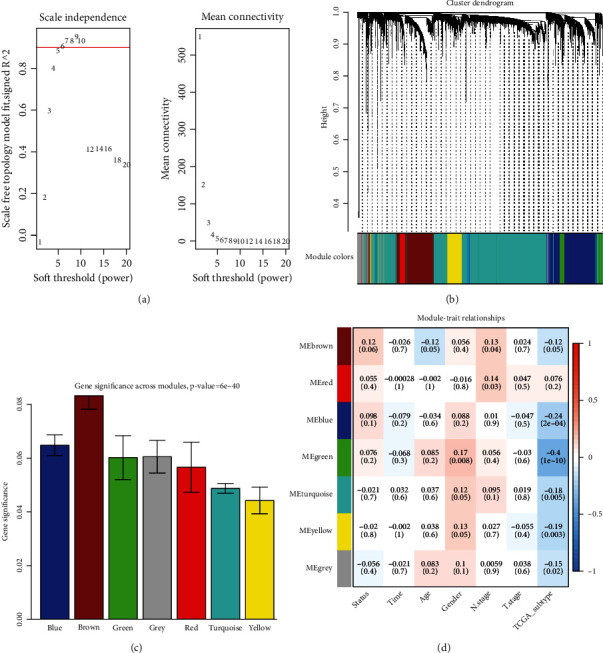
Development of coexpression network of the ncRNA expression matrix in the training set. (a) Analysis of the scale-free fit index and mean connectivity for various soft-thresholding powers. (b) The cluster dendrogram of genes. (c) Distribution of average gene significance and errors in the modules associated with survival status. (d) Module–trait relationships. Each cell consists of the corresponding correlation and *p* value, which are color-coded correlated according to the color legend.

Figure 1(a) illustrates the workflow analysis of this study. First, 2884 ncRNAs significantly linked to m6A-related genes were obtained from the TCGA dataset. The relationship between m6A-related genes and ncRNAs is shown in Figure 1(b). An aggregate of 107 m6A-related ncRNAs was obtained using the m6A2Target database and 260 m6A-related ncRNAs using the Starbase database. By merging these datasets, 3168 m6A-related ncRNAs were finally obtained (Figure 2). The gene expression was normalized for subsequent analysis. The expression matrix was divided into training and testing sets (243 and 104, respectively), and the training set was then subjected to subsequent analysis. Second, the model construction was performed using the training dataset. The coexpression network was developed utilizing the R package “(WGCNA)” for the ncRNA-related gene expression matrix and adopting 5 as the most suitable soft threshold power (Figures 3(a) and 3(b)); a total of six relevant modules were obtained. The brown and blue models were selected as key modules (Figure 3(c)). These two modules contain a total of 910 genes (Figure 3(d), *p* < 0.01).

### 3.2. Prognostic Analysis of Candidate m6A-Related ncRNAs

**Figure 4:**
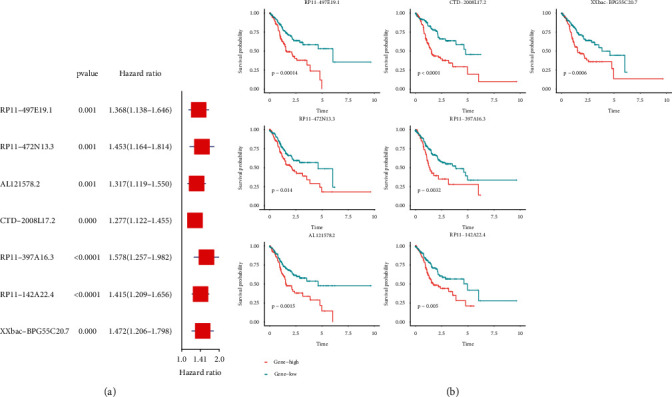
(a) Univariate Cox analysis for the expression of m6A-related prognostic ncRNAs. (b) Kaplan-Meier curve results of m6A-related prognostic ncRNAs (*p* < 0.001).

Next, the univariate Cox regression analysis was employed to select prognosis-associated genes from 910 m6A-related ncRNAs. A total of 39 candidate genes were included for further analysis. The forest plot showed that RP11-497E19.1, RP11-472N13.3, AL121578.2, CTD-2008L17.2, RP11-397A16.3, RP11-14A22.4, and XXbac-BPG5C20.7 are risk factors with hazard ratios of >1 (*p* < 0.001) in patients with GC (Figure 4(a)). The Kaplan-Meier survival curves demonstrated that high expressions of RP11-497E19.1, RP11-472N13.3, AL121578.2, CTD-2008L17.2, RP11-397A16.3, RP11-14A22.4, and XXbac-BPG5C20.7 were linked to the poor OS in the TCGA dataset (Figure 4(b)).

### 3.3. Establishing the Prognostic Risk Model Premised on m6A-Related ncRNAs

**Figure 5:**
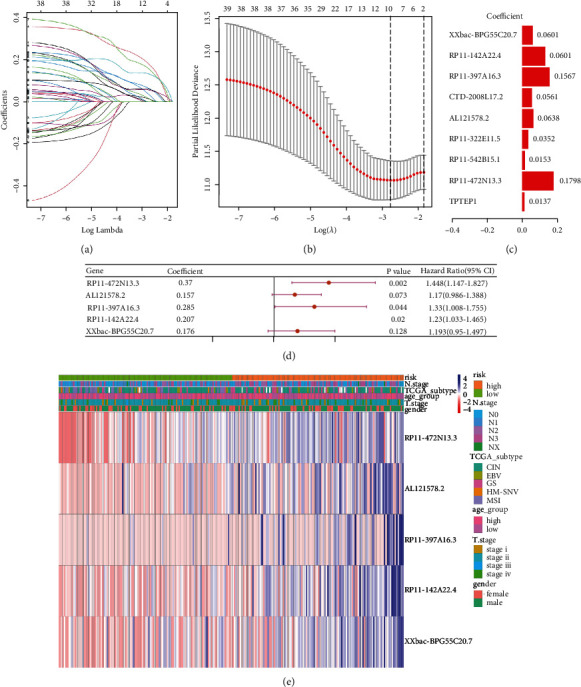
Least absolute shrinkage and selection operator (LASSO) regression was performed calculating the minimum criteria (a and b) and coefficients (c). (d) Cox values of genes obtained after LASSO regression. (e) Heatmap of risk model-related gene expression with corresponding clinical information labelled.

**Figure 6:**
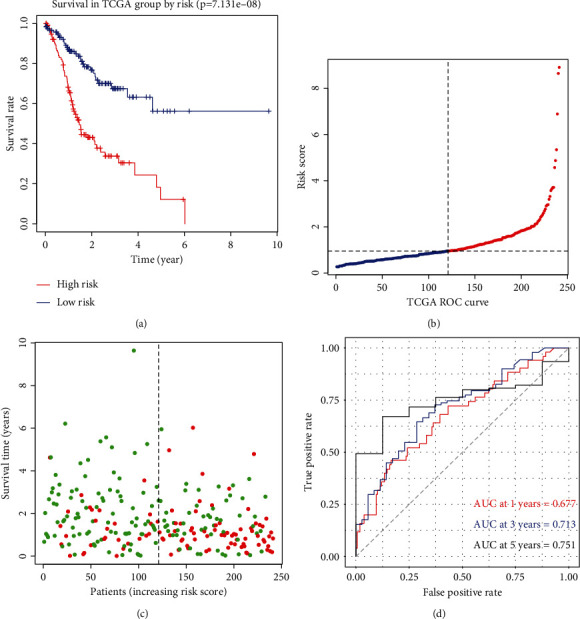
(a) Kaplan-Meier curves indicated that the high-risk groups had worse overall survival than the low-risk groups in TCGA training dataset. (b and c) The distributions of risk scores and survival status of GC patients in TCGA training dataset. (d) Receiver operating characteristic (ROC) curves of m6A-NPS for predicting the 1-/3-/5-year survival in TCGA training dataset.

The LASSO Cox analysis was executed premised on the 39 candidate m6A-related prognostic ncRNAs to establish the m6A-related ncRNA prognostic signature (m6A-NPS) (Figures 5(a) and 5(b)). The following five genes were identified: RP11-472N13.3, AL121578.2, RP11-397A16.3, RP11-142A22.4, and XXbac-BPG55C20.7. Moreover, a risk score was computed according to the coefficient for each ncRNA in all participants in the training set (Figures 5(c) and 5(d)). And Figure 5(e) illustrates the heatmap of risk model associated gene expression with the corresponding clinical information. Conversely, participants in the training group were categorized into high- and low-risk subcategories premised on the median risk scores. The Kaplan-Meier survival curve analysis revealed that clinical survival was worse in the high-risk cohort of GC patients (Figure 6(a), *p* < 0.0001). The survival status and risk score distribution in the TCGA training datasets indicated that the proportion of patients who died was considerably greater in those with high scores as opposed to those with low scores (Figures 6(b) and 6(c)). Then, ROC curves indicated that m6A-related ncRNAs hold a potential ability for predicting the OS in the training set (1-year area under the ROC curve (AUC) = 0.677, 3-year AUC = 0.713, and 5-year OS = 0.751; Figure 6(d)).

### 3.4. Validation of m6A-Related ncRNA Models in Testing Dataset and the Whole TCGA Cohorts

To verify the prognostic ability of the five m6A-related ncRNA-based models, the same formula was used in the testing cohort, each patient's risk scores were computed, and patients were categorized into the high- and low-risk cohorts premised on the median risk score. Unfortunately, no statistically significant difference in survival was observed in the testing dataset between the low- and high-risk cohorts (*p* = 7.105e − 01, Supplementary Materials Figure S1a-d). This may be a result of the low number of patients in this cohort. Thus, the same formula was utilized to generate the risk score for patients in the entire TCGA group, as it was used for TCGA training and testing datasets.

**Figure 7:**
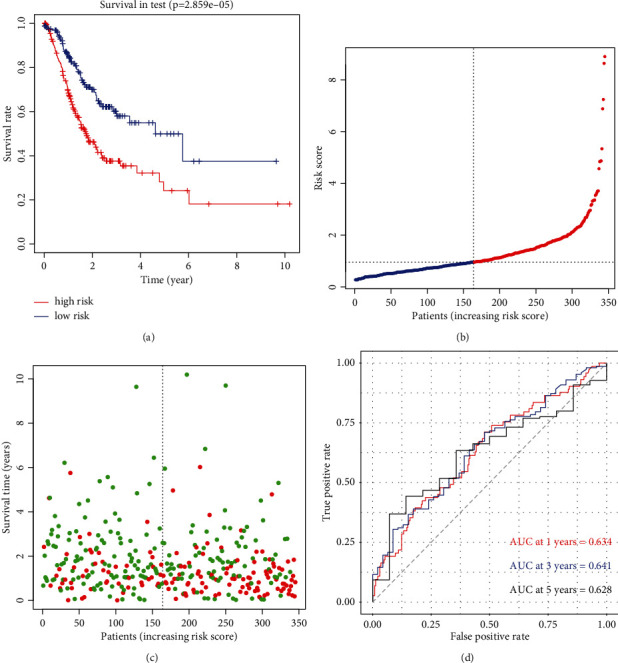
(a) Kaplan-Meier curves showed significant difference in overall survival between high and low risk groups in the whole TCGA dataset. (b and c) The distributions of risk scores and survival status of GC patients in the whole TCGA dataset. (d) Receiver operating characteristic (ROC) curves of m6A-NPS for predicting the 1-/3-/5-year survival in whole TCGA dataset.

Participants in the high-risk subcategory exhibited a poorer survival rate as opposed to those in the low-risk subgroup (Figure 7(a), *p* < 0.001). The survival status and risk score distribution in the entire TCGA datasets were consistent with that in the training set, implying that the proportion of patients who died was considerably greater in those with high scores contrasted with those with low scores (Figures 7(b) and 7(c)). Similarly, the time-dependent ROC curve analysis indicated that the model had an auspicious ability to forecast the OS for patients in the entire TCGA cohorts (1-year AUC = 0.634, 3-year AUC = 0.641, and 5-year OS = 0.628; Figure 7(d)). These findings depicted that this model may be utilized to forecast the oncologic prognosis of GC patients.

### 3.5. Prognostic Ability Assessment of m6A-Related ncRNA Model

**Figure 8:**
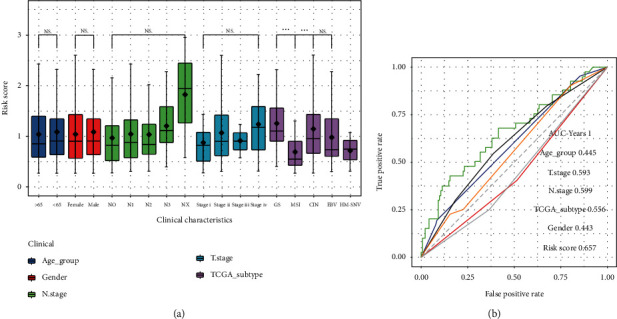
(a) Differences between risk scores in clinical characteristics. (b) ROC curves showed the predictive ability of risk score model and TNM stages.

**Figure 9:**
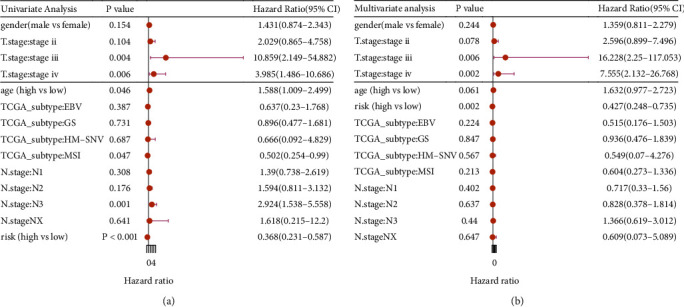
Univariate (a) and multivariate (b) Cox regression analyses of clinical characteristics and the risk score model based on TCGA.

First, risk score differences in clinical characteristics were analyzed, and the results showed significant differences only in TCGA molecular subtypes [39] (Figure 8(a), *p* < 0.001). TCGA molecular subtypes are a type of tumor classification based on molecular data and have been proven to be more clinically influential in predicting the treatment and patient prognosis as compared to the traditional histopathological classification [40]. Therefore, our results depicted that the score model was closely linked to clinical prognosis. Then, the clinical predictive power of the risk score was assessed. The ROC curve was used to characterize the predictive power of different clinical characteristics for the prognosis, and the results revealed that the risk score model had a better predictive ability for prognosis of patients with GC (Figure 8(b), AUC > 0.6). Finally, the risk score model independence was validated using the uni- and multivariate Cox regression analyses. The results implied that the model was a significant and independent prognostic factor (Figures 9(a) and 9(b); *p* < 0.001 and *p* = 0.002, respectively).

### 3.6. Functional Enrichment Analysis

**Figure 10:**
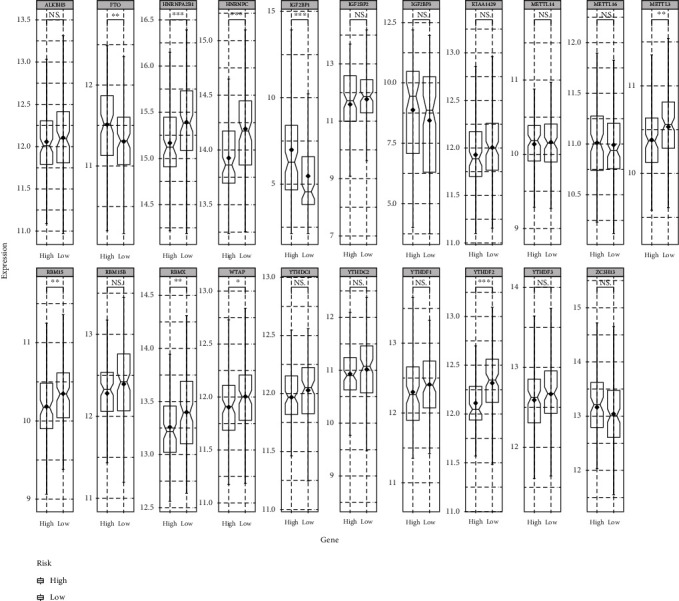
The expression profile of m6A-related genes between low- and high-risk subgroups.

**Figure 11:**
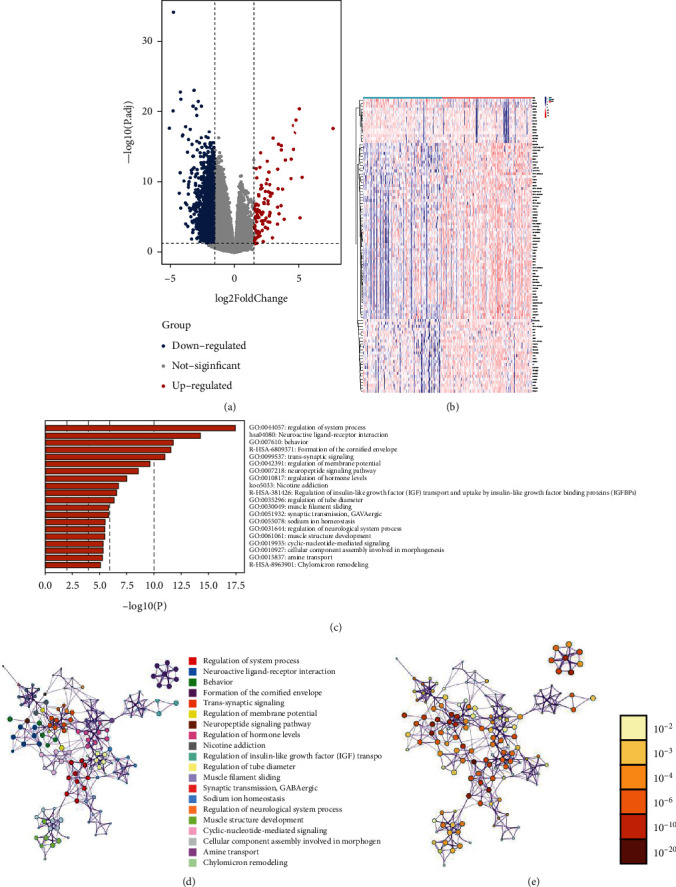
Identification of differential expressed genes (DEGs) and functional enrichment analysis. (a) Volcano map of DEGs. (b) Heatmap of DEGs in TCGA. (c) Pathway annotation of DEGs. (d and e) Pathway enrichment clustering of DEGs.

The expression analysis and difference test for 21 m6A-related genes were performed between low- and high-risk cohorts. The results revealed statistically significant differences in the expression of FTO, HNRNPA2B1, HNRNPC, IGF2BP1, METTL3, RBM15, RBMX, WTAP, and YTHDF1, of which only FTO and IGF2BP1 were considerably highly expressed in the high-risk cohort (Figure 10). These results may implicate a complex regulation mechanism of m6A regulators in the score model. Therefore, to investigate the possible pathways and biological processes in molecular heterogeneity between low- and high-risk cohorts, in the TCGA training set, 2301 DEGs were identified: 107 upregulated and 2194 downregulated genes. Then, pathway analysis of the DEGs was executed utilizing the Metascape. The results depicted that these DEGs were enriched in the following pathways: neuroactive ligand-receptor interaction, formation of the cornified envelope, IGF transport and uptake by IGFBPs, and chylomicron remodeling (Figures 11(a)–11(e)).

## 4. Discussion

In the field of epigenetics, reversible processes of m6A modifications are generally accepted to control and determine cell growth and differentiation [41, 42]. Currently, the regulatory role of m6A methylation in tumors has been attracting more attention, and in-depth genomic studies have shown that m6A modifications are closely correlated with tumorigenesis and progression [11, 27, 43]. However, the role of GC-related m6A methylation regulation based on ncRNAs is not yet fully known. We strongly believe that m6A modifications of ncRNAs play an essential role in the GC progression. Hence, potential prognostic biomarkers and therapeutic targets for GC should be identified.

In the recent decades, the progress in uncovering the genetic characteristics of various diseases has been accelerated by the high-speed development of high-throughput sequencing and bioinformatics [44]. The TCGA database, a publicly available cancer genome database, provides comprehensive cancer data, including ncRNA expression data and clinical follow-up information. Currently, TCGA data are widely used in researches related to diagnosis and prognosis of cancers [45, 46]. The present study was conducted using gene expression profile data and clinical information of GC which were extracted from the TCGA database. In this study, 347 GC samples were analyzed to uncover the prognostic value of m6A-related ncRNAs. And we performed a multistep analysis for identifying the significant prognostic m6A-related ncRNAs in GC.

WGCNA (weighted gene coexpression network analysis), an algorithm designed to characterize the gene-phenotype relationship of a given sample, avoids potential bias and subjective decisions by the unsupervised hierarchical clustering approach chosen [47]. Therefore, in this study, we applied WGCNA to analyze the m6A-related ncRNA expression dataset for the determination of networks and genes that are tightly associated with GC prognosis. Then, the univariate Cox regression was employed to determine genes with prognostic significance. The LASSO regression algorithm was used for precision and efficiency reduction dimensions of the variable selection and prognostic risk models. Finally, a novel m6A-NPS based on five ncRNAs was developed to forecast the OS of GC. Premised on the risk score, GC patients could be stratified into high- and low-risk subcategories, and the prognosis of the high-risk cohort patients was considerably poor in comparison to those in the low-risk cohort. Enrichment analysis illustrated that DEGs between the two groups regulated multiple neoplasm-related signaling pathways. Comparing the differences in clinical characteristics between low- and high-risk subcategories, significant differences in TCGA subtypes were detected between low- and high-risk subcategories, suggesting a close intrinsic association between TCGA molecular types and clinical prognosis. The univariate and multivariate Cox regression analyses demonstrated that m6A-NPS was an independent risk factor for the GC patients' prognosis. The ROC curve analysis suggested that m6A-NPS had better predictive power than other clinical parameters.

Taken together, the above results suggested that our prognostic signature based on five ncRNAs associated with m6A can be a powerful prognosticator for GC patients.

Recently, a series of studies have been focused on m6A-related lncRNA signatures to predict the clinical outcomes for gastric cancer patients. For instance, Wang et al. used TCGA database to establish an m6A-related lncRNA pair signature consisting of 25 unique lncRNAs for forecasting GC patients' OS [48]. In addition, using the TCGA dataset and the LASSO Cox regression model, Huang et al. established and identified a 14-m6A-related lncRNA prognostic signature with significant value in predicting the OS for GC patients [49]. 11-m6A-related lncRNA signature was established by Wang et al., which could independently predict the clinical outcomes of GC [50]. Up to date, there are no m6A-related miRNA signatures to be established to forecast prognosis for GC. Although the above-mentioned studies developed lncRNA signatures individually, the prognostic assessment ability of each study was different. Moreover, the ROC values of their proposed models were higher than those proposed in our study. However, there were some differences between our proposed prognostic models and these prognostic signatures. First, the perspective of our study included miRNAs and lncRNAs, not only screened lncRNAs. Second, in consideration of the value of future clinical applicability, the number of ncRNAs in the risk model should be as small as possible, but the number of lncRNAs they previously established was much higher than the number in our 5-ncRNA signature.

In this study, 39 m6A-related prognostic ncRNAs were identified in 347 patients with GC. Finally, five of them were determined to develop m6A-NPS. As far as we know, this study first established the role of m6A-NPS in GC, namely, RP11-472N13.3, AL121578.2, RP11-397A16.3, RP11-142A22.4, and XXbac-BPG55C20.7, also known as lnc-ARHGAP12, lnc-HYPM-1, lnc-WDR7-11, LINC02266, and lnc-PRIM2-7, respectively. Five ncRNAs are all classified as lincRNAs and have not been preliminarily investigated to date because only a few reports have evaluated the interaction of ncRNAs with m6A-related genes. However, our findings might still help identify prognostic ncRNAs that can be potentially targeted by m6A regulators, thus offering understandings of their probable functions in gastric carcinogenesis and progression.

Despite the robust prognostic signature of five-ncRNA established in this study. We must acknowledge that this research has several limitations. First, our results were obtained and validated using the TCGA dataset. Since no applicable external dataset was available for validation, internal data validation was merely performed. Therefore, an independent GC cohort with larger sample size should be utilized to further confirm our prognostic ncRNA signature. Second, further cell line and animal functional experiments are required, and the intrinsic mechanisms of prognostic ncRNAs as well as their mutual interactions to m6A-related genes should be further investigated.

Finally, whether the combination of 5-ncRNA prognostic signature with other clinical features could potentially improve the predictive power remains an important question for our future studies.

## 5. Conclusions

In this study, we performed integrated bioinformatics analyses to determine prognosis-associated ncRNAs and identified five m6A-related ncRNA signatures to forecast the OS in GC patients.

## Data Availability

The raw data of this study are derived from m6A2Target database (http://m6a2target.canceromics.org/) and the TCGA database (https://portal.gdc.cancer.gov/), which are publicly available databases.

## Supplementary Materials

Supplementary Materials Figure S1: (a) Kaplan-Meier curves showed no difference in overall survival between high and low risk groups in the TCGA testing dataset. (b and c) The distributions of risk scores and survival status of GC patients in TCGA testing dataset. (d) Receiver operating characteristic (ROC) curves of m6A-NPS for predicting the 1-/3-/5-year survival in TCGA testing dataset.
