# Supplementary figures and images for: Identification of Five N6-Methylandenosine-Related ncRNA Signatures to Predict the Overall Survival of Patients with Gastric Cancer
Source: Dis Markers. 2022 Apr 8;2022:7765900. doi: 10.1155/2022/7765900 (PMC9239763; doi:10.1155/2022/7765900)

Survival in test ( $p=7.105e-01$ )

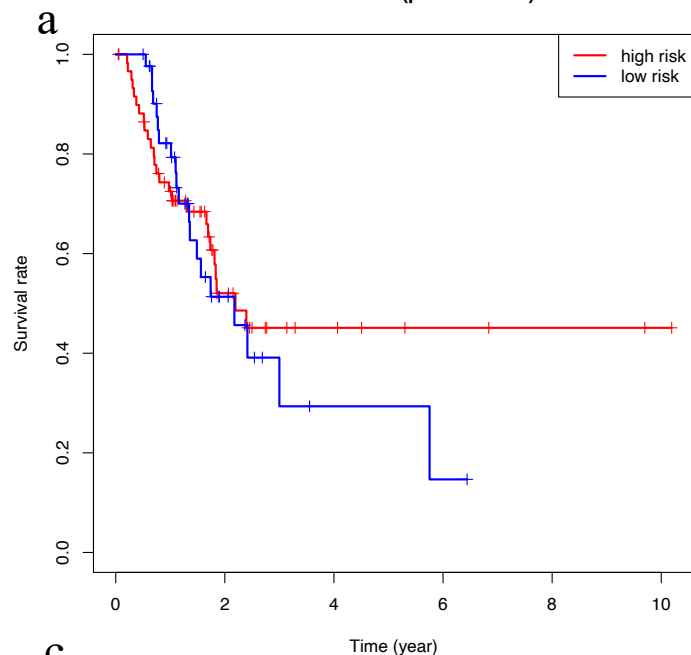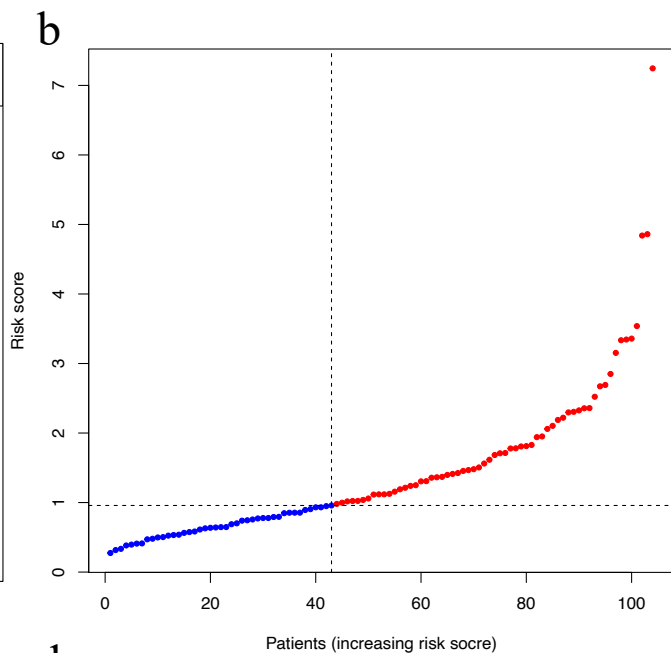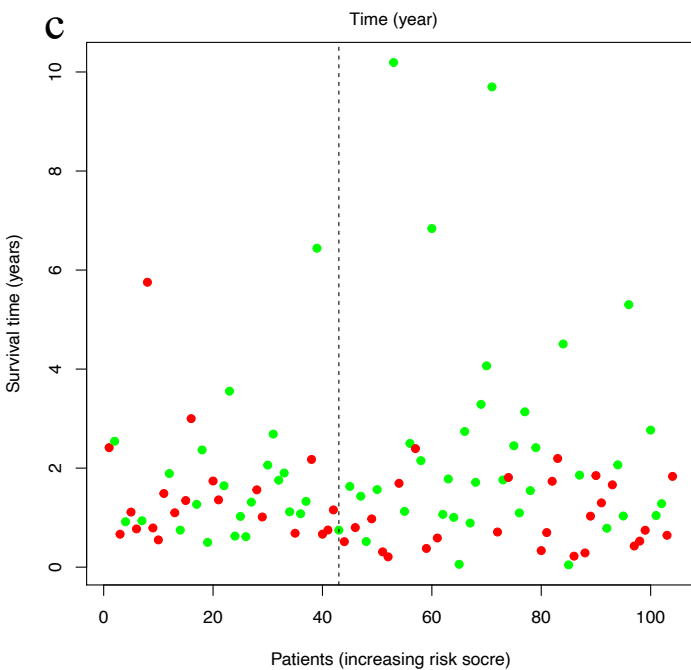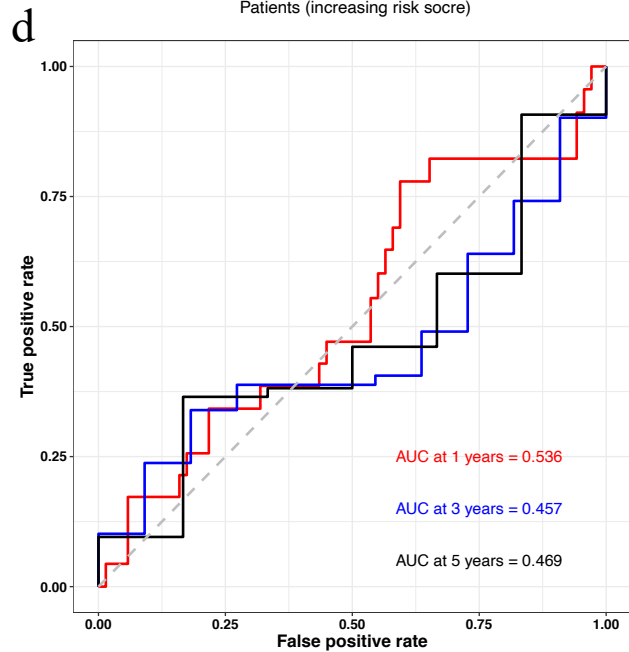

Supplement: Supplementary Materials — Figure S1: (a) Kaplan-Meier curves showed no difference in overall survival between high and low risk groups in the TCGA testing dataset. (b and c) The distributions of risk scores and survival status of GC patients in TCGA testing dataset. (d) Receiver operating characteristic (ROC) curves of m6A-NPS for predicting the 1-/3-/5-year survival in TCGA testing dataset. [file 7765900.f1.pdf]
